# Open-source framework for detecting bias and overfitting for large pathology images

**DOI:** 10.1371/journal.pone.0341715

**Published:** 2026-02-19

**Authors:** Anders Sildnes, Nikita Shvetsov, Masoud Tafavvoghi, Vi Ngoc-Nha Tran, Kajsa Møllersen, Lill-Tove Rasmussen Busund, Thomas K. Kilvær, Lars Ailo Bongo

**Affiliations:** 1 Department of Computer Science, UiT The Arctic University of Norway, Tromsø, Norway; 2 Department of Community Medicine, UiT The Arctic University of Norway, Tromsø, Norway; 3 Department of Clinical Pathology, University Hospital of North Norway, Tromsø, Norway; 4 Department of Medical Biology, UiT The Arctic University of Norway, Tromsø, Norway; 5 Department of Clinical Medicine, UiT The Arctic University of Norway, Tromsø, Norway; Chengdu University of Traditional Chinese Medicine Wenjiang Campus: Chengdu University of Traditional Chinese Medicine, CHINA

## Abstract

Even foundational models trained on large-scale datasets may learn to rely on non-relevant artifacts such as background color or color intensity, leading to overfitting and/or bias. To ensure the robustness of deep learning applications, there is a need for methods to detect and remove the use of these artifacts. Existing debugging methods are often domain- and model-architecture-specific, and may be computationally expensive, hindering widespread use. We propose a model-architecture-agnostic framework to debug deep learning models. To demonstrate the utility of our framework, we test it using a widely used dataset from histopathology, which has been tested in other literature. The dataset features very large images that typically demand large computational resources. We demonstrate that the framework can replicate known bias patterns in a pre-trained foundation model (Phikon-v2) and a self-trained self-supervised model (MoCo v1). Our framework contributes to the development of more reliable, accurate, and generalizable models for WSI analysis, and is available as an open-source tool integrated with the MONAI framework at https://github.com/uit-hdl/feature-inspect.

## Introduction

Examining tissue specimens under a high-powered microscope remains the gold standard for cancer diagnosis by pathologists. Currently, glass slides are digitized into whole-slide images (WSI), each potentially having billions of pixels and millions of cells. However, it is difficult for humans to identify and use all prognostically relevant features in a WSI. Therefore, deep learning (DL) models show great promise for WSI analysis both as a standalone tool and as decision support for pathologists. For example, DL has demonstrated its usefulness for cancer type classification [1,2], tissue segmentation [3,4], cell segmentation [5,6], and analysis of tissue microenvironments [7,8].

An important limitation for WSI model development is the lack of annotated datasets [1]. Consequently, self-supervised learning (SSL) methods trained on larger, unannotated datasets have recently been used for WSI analysis [9,10]. A popular SSL approach for natural image prediction is contrastive learning (CL) [11,12]. However, many contrastive learning models suffer from low generalizability [13,14]. This means models are overfitted to their datasets, which in turn can lead to lower and unpredictable sensitivity and specificity on unseen data. This is an especially important limitation for medical applications where variability in data, such as differences in staining protocols, scanning equipment, or patient populations, is common. Overfitting in this context can hinder the adoption of these models in clinical practice. This can also make models biased since they rely on non-clinical features to make their predictions. Lack of generalizability in contrastive learning models may arise from their tendency to rely on shortcuts. Shortcuts occur when a model identifies and uses dataset-specific patterns or features, known as artifacts, to make predictions. These artifacts are typically non-generalizable and may be unique to certain datasets. Ideally, models should avoid learning artifacts, as these are often dataset-specific, lack clinical relevance, and can lead to overfitting and unreliable predictions.

**Fig 1:**
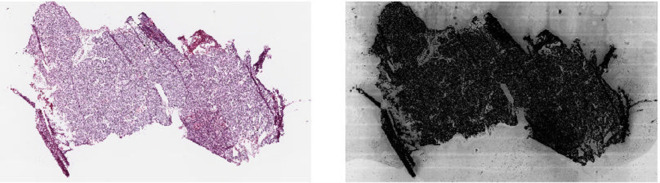
Hidden artifacts in WSIs: WSI from TCGA (left) and a modified version (right) contrast enhanced with equalized histograms and stripped of colors. This reveals scanner stripes and air bubbles, not usually visible to humans.

Artifacts can be introduced during tissue preparation, imaging, and staining equipment, or through the chemicals used [15–18]. These include uneven or inconsistent staining, tissue folds, wrinkling or tearing, variation in section thickness, scanning focal blur, and scanner-specific color profiles. These can affect human pathologists too, which is why there are extensive guidelines available for slide validation (see for example [19]). However, whereas humans may not see or overlook some of these artifacts, deep learning models may learn to use them. Examples of artifacts that are difficult to see for humans, but that a DL model can use, are shown in Fig 1. Artifacts can be uniquely present on a single slide or occur in sets processed in batches. Batch effects are widely known and accounted for in other disciplines such as bioinformatics [20], but have only recently received attention in computational pathology [17,21,22]. For example, [23] showed that some institutions had proportionally more patients with severe cancers, and that models could identify these sites and use site-specific artifacts as shortcuts for predictions. The result is that a model may misdiagnose a patient, giving the most common diagnosis rather than considering relevant clinical data. They also show that common color augmentation and normalization techniques were insufficient to prevent models from learning these artifacts. [24] did a similar experiment on 8579 slides from The Cancer Genome Atlas (TCGA) datasets and showed that with minimal fine-tuning or re-training, a model could map a tile from a slide to the institution where the slide was produced with approximately 80% overall accuracy. Both studies advocate pertinent consideration when sampling and using WSI datasets for deep learning. This underscores the need to systematically evaluate model overfitting and data bias. And without careful inspection, there may be unforeseen consequences: [25], for example, found that a DL model for COVID screening of x-rays had the same performance when removing most of the relevant (lungs) tissue from the images, showing that the model was relying on information from just the borders of the image as a shortcut. [26] found that an algorithm used in several US health care systems would assign similar risk scores to black and white patients even when the black patients were much sicker, meaning that white people were more likely to get earlier treatment. As more hospitals begin using AI, these examples highlight a need to standardize additional testing of models, so that everyone who makes a DL model, especially for clinical use, can ensure that end users/patients receive fair treatment.

Using large amounts of data to train deep learning models may seem like an intuitive way to prevent shortcut-learning, as models have to generalize more when looking at larger datasets. Foundational models are often trained on datasets that are orders of magnitude larger than those used for conventional SSL models. There are increasingly many of these models available, and they have been popular since they have overall good performance for a range of tasks, but it has been found that several foundational models are not immune to batch-effects and shortcut learning. [27] tested nine foundational models trained on WSI data and found that they could detect tissue source sites in TCGA, indicating considerable batch effects in the models.

Finding the right test for bias and overfitting is challenging. Tests introduce computational overhead, take time to implement, and interpretation can be subjective. Each model architecture may be different, so testing multiple models introduces additional overhead. In related work, it is common to select one or two methods, even though there may be other relevant tests for WSI models. Type of evaluation methods include, but are not limited to, heatmaps [28–30], vector dimensionality reduction (DR) plots [21,23,31], external validation datasets [32], loss curves [33], linear probing [23,24,27], and estimating domain boundaries [34,35]. While increasing the number of tests could enhance error detection, the need for extra computational resources and/or data (if using external validation) also makes it impractical. Therefore, we believe there is a need for a general testing framework that scales to large WSI datasets and that can be readily used for multiple models. Our contributions are threefold. First, we introduce a framework with two key features:

1. **An intuitive framework for detecting and analyzing bias** - We provide user-friendly tools to visualize and assess how batch effects influence model performance and contribute to overfitting in deep learning models. This allows researchers to identify and mitigate potential biases more effectively.
2. **model-agnostic** methods that can handle the large volumes of data needed for WSI datasets.

The framework uses vector DR plots and linear probing. Both methods are typically used to explore task-specific model classification, but can also be used to explore batch effects in models. Since they work with embeddings, they can in theory work with any deep learning model architecture that produces a vector of features (such as convolutional neural networks and vision transformers). This article focuses on histopathology WSIs, but other types of image-data can also be used.

Our second contribution is to demonstrate the framework using two different models. We start by replicating a part of the study of [27] and use the foundational WSI model Phikon-v2 [36] to evaluate tissue-source site (TSS) (also known as acquisition site or institution) biases in TCGA. We focus on TSS because prior work has shown TSS to be a dominant, learnable confounding factor in WSIs, particularly from TCGA. This applies to both regular convolutional models trained on smaller datasets and to foundational models [22,27]. We also test a model that we trained from scratch on a single dataset. We chose MoCo v1 [37], a CL SSL model with Inception-V4 [38] as the encoder and decoder. MoCo v1 was also used by [21] to detect slide-level biases, making it a relevant choice for detecting and understanding potential batch effects in our study.

The third contribution is making the framework scalable for consumer-grade GPUs, offering easy integration with cuCIM for fast UMAP computation, and providing code that can be integrated into training loops in the popular open-source Medical Open Network for Artificial Intelligence (MONAI) [39] framework. The MONAI framework wraps PyTorch [40] models with extensions that make the framework appropriate for the scale and testing needed for medical data such as WSIs. Doing model inspections during training allows users to track model bias over time, which can serve as an early-stopping mechanism (using threshold values for linear probing or UMAPs) or as a debugging tool to understand learning behavior. Furthermore, since MoCo v1 and many other SSL models consume a lot of VRAM, we also demonstrate the use of sequential checkpointing to reduce VRAM usage. Such optimizations enable debugging on consumer-grade hardware, eliminating the need for expensive computers to inspect models.

We demonstrate the tool on a single dataset to illustrate its use and engineering trade-offs, not to conduct an exhaustive multi-cohort survey. Our examples highlight some of the challenges when analyzing bias for models trained on WSIs: scoring functions that are influenced by data heterogeneity, limited sample sizes, and limited resources (VRAM). The main focus is to create a modular, easy-to-use software to address the core challenges of developing robust, unbiased deep learning models for WSI analysis. By optimizing and standardizing model training processes and doing systematic evaluation, we facilitate the generation of more reliable, accurate, and generalizable models for WSI analysis. The framework is open source under the Apache 2.0 license and available at https://github.com/uit-hdl/feature-inspect.

### Related work

solo-learn [41] is an open-source framework that streamlines the development of self-supervised learning (SSL) methods for visual representation learning. It offers a modular PyTorch-based codebase for training, evaluation, and fine-tuning of SSL models and includes utilities for representation analysis, such as UMAP visualizations and linear probing. solo-learn emphasizes high-throughput data pipelines (for example, NVIDIA DALI [42]) to accelerate I/O and augmentation in large-scale SSL experiments. Their framework differs from ours: our APIs are intended to interface only with embeddings, not a selection of models, and we provide an exploratory view of UMAPs and integrations with MONAI to simplify adoption within medical-imaging pipelines.

[22] introduce and evaluate a “Robustness Index” to assess TSS-awareness in embedded space. The metric measures the distance between points in embedded space, taking into account how many of their neighbors are from the same medical center, as well as given clinical variables such as tissue type. Their analysis of ten publicly available pathology foundation models confirms previous findings that most models’ embeddings are strongly organized by TSS rather than by biological class.

Alternatively, data quality assessment can be performed at the input level rather than by examining model features. Tools such as HistoQC [17] systematically detect common slide-level artifacts and outliers (for example, excessive background, staining failures, tissue folds) and are useful as a preprocessing step to reduce and identify obvious, low-level sources of bias prior to model training. Note that HistoQC and similar input-level tools do not replace embedding-level checks: some TSS artifacts are subtle and only become apparent when inspecting learned representations or probing for separability.

## Material and methods

### Framework for inspecting embeddings

In this section, we describe the design and implementation of our proposed framework for inspecting model features of DL models. We then show an example workflow using the framework for bias and overfit-detection on a WSI dataset.

#### Architecture and design.

**Fig 2:**
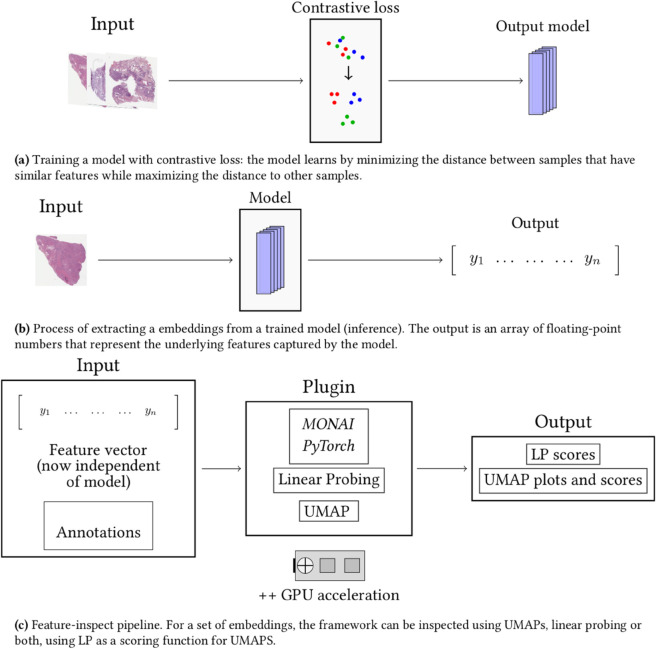
Pipeline overview. (a) Training a model with contrastive loss: the model learns by minimizing the distance between samples that have similar features while maximizing the distance to other samples. (b) Process of extracting a embeddings from a trained model (inference). The output is an array of floating-point numbers that represent the underlying features captured by the model. (c) Feature-inspect pipeline. For a set of embeddings, the framework can be inspected using UMAPs, linear probing or both, using LP as a scoring function for UMAPS.

The architecture for our framework (Fig 2) is designed to work on a consumer-grade computer with a single GPU. It can be used during DL model training or to evaluate a pre-trained model. Our framework is available as a Python API, which is invoked with a few lines of code. To evaluate a model, users must have a trained model, a dataset, and labels which could either be clinically relevant (for example tumor prognosis) or related to bias analysis (for example TSS).

Testing begins by extracting features using inference with a DL model (Fig 2b). The features are embedded representations of the input. In SSL models, these are typically vectors represented by approximately 1000 floating points. The inference output is used as input to the bias/overfit detection module (Fig 2c). Users supply a set of either sensitive or prognostic variables for each embedding. For example, to explore potential gender bias, users can assign images/embeddings with gender labels. The output from the bias/overfit detection can be saved to a text file or, for UMAPs, rendered as an interactive website for visual exploration. After inspection, a model developer can consider using different models or implementing techniques such as regularization, data cleaning, or data weighting to make the model more robust to unseen data.

For our linear probe and model training, we use MONAI. It is a widely used PyTorch extension for medical data. It includes tools for image preprocessing, augmentation, and analysis, along with optimizations such as distributed processing of models and data, smart caching, and improved image processors (such as cuCIM from [43]). Additionally, MONAI integrates with tools such as TensorBoard [44], making it easy to track model loss, validation accuracy, and other metrics in a web interface. MONAI also includes other built-in interpretability tools, such as heatmaps and occlusion maps, that can be used alongside the tools we provide in our framework.

#### Bias detection workflow - A demonstration.

We demonstrate our framework through a case study that identifies potential biases and overfitting in a DL model. A minimal workflow is detailed in Algorithm 1. First, the training data and annotations are loaded from a specified directory (step 01). The dataset consists of WSI tiles in JPG format with slide-level labels indicating the institution of origin. Embeddings are extracted after a forward pass of the model. Steps 03 and 04 produce UMAP plots and linear probing results. Results can be printed to a console, rendered to TensorBoard, or written to HTML. Algorithm 2 has a MONAI training loop for a simple, supervised model. To avoid slowing down the training loop too much, UMAP and linear probing can be configured to only run for a given epoch interval. This allows users to visualize the training process over time.

**Algorithm 1 General workflow of our framework for inspecting and analyzing models trained on a given dataset.**



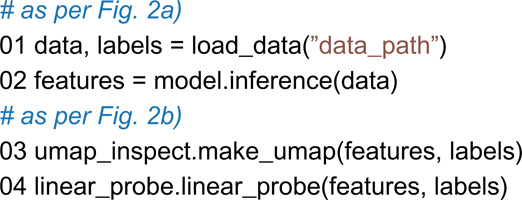



**Algorithm 2 Outline for how to integrate the feature-inspect tool for a classical MONAI model training loop.**

The code in the “val_handlers” are the only additional lines of code needed, the rest are common configuration options for the MONAI training setup. “dl” is short for “dataloader”. For more information, see MONAI documentation at https://docs.monai.io/en/stable/engines.html.

evaluator = SupervisedEvaluator(

 val_data_loader=dl_val,

 network=model,

 val_handlers=[

  feature_inspect.UmapExplorer(every_n=20),

  feature_inspect.LinearProber(every_n=20)])

trainer = SupervisedTrainer(

 max_epochs=epochs,

 ValidationHandler(1, evaluator),

 train_data_loader=dl_train,

 network=model,

 inferer=SimpleInferer())

trainer.run()

### Overfitting and bias detection methods

#### UMAP plots for qualitative analysis.

Uniform Manifold Approximation and Projection for Dimension Reduction (UMAP) [45] plots scales well to large inputs and offers a visually appealing interface for data exploration, aiming to identify data anomalies, wanted or unwanted clusterings, and more. UMAP plots have become common for showing that a model is more fair or less biased [13,46], also in histopathology [7,21,23,28,31,47].

UMAP computations are generally fast, though CPU-based implementations can take several minutes for millions of samples. A UMAP is made by constructing a graph of high-dimensional data, then using a stochastic gradient descent to minimize the difference between the high- and low-dimensional representations. The original UMAP algorithm is executed on CPU(s) [48], but several libraries offer GPU-optimized implementations. [49] have published a GPU-based implementation in the “cuml” package [50]. Their speedups for GPU compared to a naïve CPU implementation were up to 100x, depending on the data and hyperparameters. We use this as the default option for computing UMAPs in our framework.

**Fig 3:**
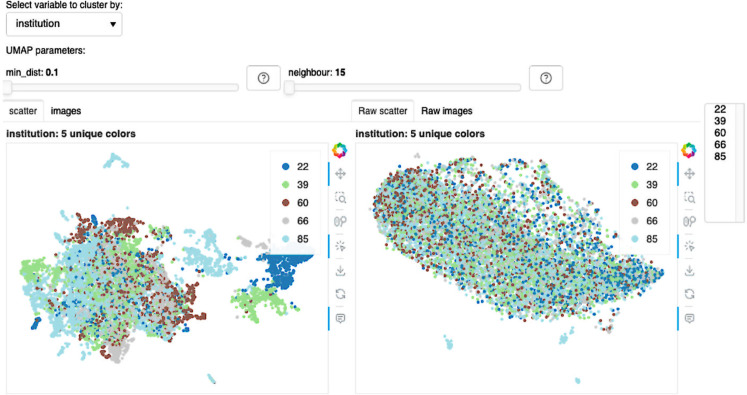
UMAP web user interface: The top-left drop-down menu allows the user to select a variable to cluster by. Below are two common UMAP parameters that the users can drag and select to see different UMAPs below. The plots have common interaction tools such as zooming and selecting regions. To the right, there is a list to hide or show specific classes. The left UMAP plot shows a UMAP from embeddings, and the right shows data from the same tiles, but only using the raw pixels.

We have built a web interface that allows a user to quickly navigate through UMAP plots (Fig 3). The web interface makes it easy to share results and enable data exploration. We embed UMAP plots with different parameters together and allow side-by-side viewing with embeddings from raw data. Plots from raw data can help reveal whether the model is doing clustering on relevant features or merely picking up obvious patterns such as large shapes or color intensities. We also provide several scoring functions that can be used to assess the quality of a UMAP plot. Silhouette scores [51], a metric for clustering quality, are commonly used. We also provide “k-nearest neighbor” (KNN) and Spearman correlation (CPD) from [52], metrics to measure the quality of local versus global structure preserved in the plot from the original high-dimensional embeddings.

For a thorough analysis, multiple UMAP plots should be generated. There is no guideline for how many points are needed to debug a model. Using fewer data samples can enhance interpretability by making patterns easier to observe, but these patterns may also arise from the choice of data used. Using many points can reveal global patterns, but can also hide smaller anomalies as noise. Therefore, doing multiple plots with different sample sizes is recommended for debugging.

#### Linear probing for quantitative analysis.

**Fig 4:**
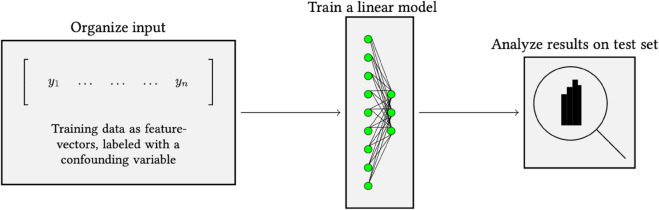
Linear probing: Embeddings from inference are labeled and then trained with a linear layer. The linear layer has maps from n to m neurons, where n is the number of features from the model, and m is the number of classes to predict. The result will be the output neuron with the highest score, which can be used to measure prediction accuracy.

For UMAP interpretation, we build a scoring function inspired by [23,24], and [27]. They used linear probing (LP) (Fig 4) to identify whether a model was able to predict tissue-source site from WSIs in DL models. The main idea is that the TSSs may have either hidden or visible artifacts, such as specific dye colorings or scanner imprints, that models learn to recognize and use. The linear probing is an easy way to test for the presence of such artifacts.

To perform linear probing, [24] used models pre-trained on either WSIs or the ImageNet dataset [53]. They froze all the model weights, but added new layers at the end of the neural network. Freezing all the weights and attaching layers is equivalent to extracting the embeddings once and training a separate linear model. Hence, this technique is model-agnostic, as long as the model operates on latent-space representations. The attached linear layers were trained for up to five epochs with the same input, now using TSS as a label. The final output layer would therefore have *n* neurons, where *n* was the number of TSSs. Their overall accuracy was high, ranging from 60% to 80% across different TCGA datasets. This scoring function is therefore similar to a UMAP - in the sense that we reduce from high to low dimensions - but it only provides a single number, accuracy, as an output.

Linear probing is quick to compute and intuitive to interpret. Overall memory usage is low because no intermediate data is stored in the LP model, and embeddings extracted from it typically have only around 1000 floating-point values each. A batch of 256 embeddings extracted from Inception-V4 uses approximately 1 GB of VRAM, which should be possible to accommodate on most computers. To speed up LP computation, users can select a limited number of samples/tiles from their test data. However, as with UMAPs, there is no best practice for selecting data for linear probes. Limited samples per class can lead to underfitting and affect LP scores. [24] split TCGA into two groups and trained separate two-layer linear probes: one on institutions with the most tiles (76% TSS accuracy) and the other on those with fewer slides (56% accuracy). These results suggest that LP accuracy scores are influenced by the number of samples per class. This also means LP scoring can be difficult to use with some types of variables. For example, [21] also investigated slide-level biases in their self-supervised contrastive learning model. They believed their model was learning slide-level features, such as blurring and pen-level markers, similar to the TSS bias we investigate in this paper. This would not be a simple task for linear probes since there are many different classes with a limited number of samples per class.

### Framework validation and performance optimization on consumer-grade GPUs

This section presents the use case and the performance optimization techniques applied in our framework.

#### Use case: TSS-level bias in TCGA.

[23,24,27] all investigate tissue source site (TSS)-level bias in models trained on the datasets from The Cancer Genome Atlas [54]. If a model is able to detect TSS, it may make similar predictions for all samples within the same TSS, based on the most common clinical variable(s). This builds on the findings from [55] that show how outcome and survival vary across TSS. For example, [23] found that false-positive detections were much higher in a TSS from TCGA’s breast cancer dataset, which had a majority of patients with African ancestry. [23] also found that there were measurable differences in the TSSs in TCGA by looking at color entropy, kurtosis, contrast, and more.

We use the Lung Squamous Cell Carcinoma (LUSC) WSI dataset [56] from TCGA. This dataset was chosen because it was used in similar work with bias investigation [23,24,27] as a representative dataset with TSS artifacts. The LUSC dataset contains 1100 tissue slides from 495 patients across 35 institutions. Of these, 753 slides have tumors and 347 are normal. All the slides were scanned by the Aperio scanner model. The tissue slides are scanned at 20x magnification and fixated using freezing. Data is collected from multiple tissue source sites, but all should adhere to the same guidelines, documented on the TCGA website [57].

Similar to [27], we ranked each TSS by the number of tiles, and selected tiles from the top five TSSs for investigating TSS bias. Restricting the analysis to the five largest TSSs ensures sufficient samples per class for reliable linear probe training and for visualizing structure in UMAP embeddings. Our ranking was done *after* filtering out tiles with high whitespace content. The top five institutions were “22” (Mayo Clinic - Rochester), “39” (MSKCC), “60” (Roswell Park), “66” (Indivumed), and “85” (Asterand), each with 19499, 32686, 19300, 25694, 49355 tiles, respectively. In addition, we balanced each TSS such that tiles were drawn from comparable distributions of slides across tumor stages. This step was taken to minimize clinical differences between TSSs, making it more likely that observed patterns or scores reflect TSS-bias rather than relevant clinical variation. Since only TSS “66” had patients with stage IV cancer (2.56% of patients), we only used slides from cancer in stages I, II, and III.

#### SSL model trained on a single dataset.

To investigate TSS biases in TCGA, we train a CL model with a similar architecture to that in [21], which investigated slide-level biases in CL models. They used a MoCo v1 [37] model with Inception-V4 [38] as encoder and decoder. The Inception-V4 encoder is used for downstream classification tasks. Inception-V4 is a convolutional neural network that uses residual connections to learn features. The output layer has 1536 neurons. Each WSI is preprocessed by tessellating into 1024x1024 tiles with 25% overlap. Tiles with more than 85% whitespace are removed. Each tile is then downsampled to 299 x 299 pixels and color-normalized using the Vahadane method [58] with a reference image from TCGA, chosen by [21] for its clear staining and well-defined cell structure. The reference image is available both in [59] and on our GitHub repository: https://github.com/uit-hdl/code-overfit-detection-framework. We also use image augmentations during training:

- cropping regions of the image, with a scale from 20% to 100% of the image
- 80% chance to add color jitter to the image, with adjustments of 40% brightness, 40% contrast, and 1% hue.
- 20% chance to add grayscale to the image
- 50% chance to horizontally flip the image
- 50% chance to vertically flip the image

For training MoCo v1, we use a 70-15-15 split between the training, validation, and test sets. The training was performed on all slides available in TCGA-LUSC, not just the top five TSSs. The number of tiles per split is given in Table 1.

**Table 1:** Data splits used for training on TCGA. Slides from one patient can only occur in one split. This can result in a different number of tiles for each train/validation/test stratification. The numbers are therefore averaged from three runs. *n* refers to the number of tiles. *n*_5_ is the number of tiles after balancing (taking an equal number of samples) from the top 5 TSS. $n_{5}^{stage}$ is the number of tiles from *n*_5_, after an equal number of tiles per tumor stage (I, II, and III).

Split set	Amount of samples in %	*n*	$n_{5}$	$n_{5}^{stage}$
train	70	189322	67655	49480
validation	15	23666	14497	10602
test	15	50621	14498	10602
total	100	264609	96650	70686

#### Reducing memory usage: Sequential checkpointing.

A significant challenge in training an SSL model is having sufficient computational resources. One of the primary constraints is the amount of GPU memory, known as VRAM. Today’s consumer-grade computers typically feature a single GPU with up to 24 GB of VRAM. This is not enough to load several foundational or SSL models and therefore hinders consumers from fine-tuning, inspecting, or re-training a model. For our MoCo model, we were limited to a batch size of 64 on a GPU with 32 GB of VRAM.

**Fig 5:**
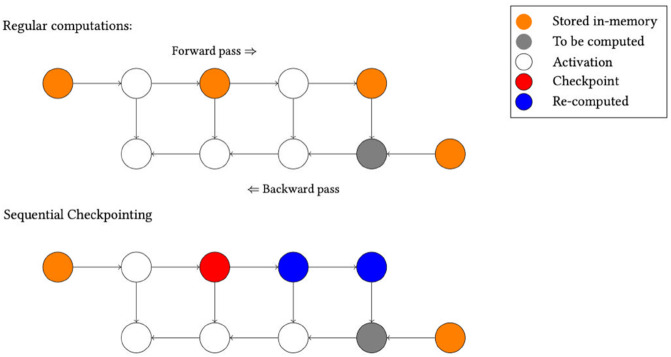
Sequential checkpointing: In a regular computation (above), information from the forward pass of the model is stored in memory. To do a backwards pass, this information is passed on for calculations. For sequential checkpointing (lower part), not all information is kept in memory. Therefore, to get the information needed for the gray node, the latest checkpoint is found (red), and information from that checkpoint is recalculated (blue nodes).

To reduce GPU memory usage in MoCo v1, we implement sequential checkpointing [60]. It divides the forward pass results into checkpoints, reducing memory usage. Conventional training stores all intermediate values for fast backpropagation, but consumes *O*(*n*) memory for a network of depth *n*. In contrast, checkpointing reduces memory consumption by only storing information in some nodes, but it increases computational overhead to a quadratic run-time due to full forward-pass recalculations. Fig 5 illustrates regular training and training with checkpoints. As in [61], we set the number of checkpoints to be $\sqrt{n}$. So for MoCo v1, we use four checkpoints, which allows us to store a batch size of 128 tiles in 10 GB of VRAM, a reduction of more than half.

#### Phikon-v2 foundational model for histopathology.

For TSS-level evaluation, we use Phikon-v2 [36], a DinoV2-based model with 307 million parameters [12]. It is trained on 460 million pathology tiles at 20x magnification from public datasets, including TCGA. The tiles are filtered using a privately trained bi-directional U-Net [62] model to exclude background and artifacts in tiles. Phikon-v2 learns using a “student-teacher” distillation loss. It is trained for 250 000 iterations using a flexible learning rate schedule and an Adam [63] optimizer. Phikon-v2’s large size and dataset make it ideal for illustrating batch effect detection and evaluating the scalability of our framework.

#### UMAPs to detect TSS.

To detect TSS biases using UMAP plots, we use two sets of randomly sampled tiles. The first has 10 000 tiles to detect local patterns, and the second has 70 000 tiles to detect global patterns. The tiles are labeled by TSS. We use the default UMAP hyperparameters from the authors [45], which have set “min_dist” to 0.1 and “n_neighbors” to 15. These defaults are intended as generally applicable starting points and may be tuned for a given dataset [64]. Conceptually, n_neighbors governs the balance between local and global structure (smaller values emphasize local neighborhoods; larger values promote global coherence), while min_dist controls how tightly points may be packed in the low-dimensional space (smaller values permit compact clusters; larger values enforce more separation). For bias detection, our primary objective is to reveal unwanted, large-scale groupings by TSS; thus, one might increase n_neighbors to accentuate global structure, while keeping min_dist small to maintain cluster visibility. Although prior work has proposed heuristic settings in other domains (for example, 1% of the sample size for single-cell data [52] and 3-10% in related analyses [65]), establishing optimal hyperparameters for histopathology bias/overfitting is beyond the scope of this study. Accordingly, we report results using the defaults of [45] and provide an interactive, web-based viewer with sliders for n_neighbors and min_dist to enable the reader to perform systematic sensitivity analysis.

#### Training a linear probe for TSS classification.

To evaluate whether models can identify tissue source sites from tiles alone, we train a linear probe on embeddings extracted from our Phikon-v2 and Inception-V4 encoders. The split configuration for training the linear probe on TCGA-LUSC are in Table 1. We trained the LP for 20 epochs using the Adam optimizer [63]. 95% confidence intervals were estimated by bootstrapping (randomly sampling the test-set with replacement) 100 times. We also compute and average Cohen’s κ [66] from the accuracy values.

#### System environment.

We run our code on a consumer-grade PC with an Intel i9-11900K processor, an NVIDIA RTX 3090, and 128 GB of RAM. For testing of sequential checkpointing, we also used an HPC computer with Intel(R) Xeon(R) Platinum 8358 CPU and an NVIDIA Tesla L40S with 46 GB of VRAM and 2 TB of main memory. We use PyTorch version 2.8, MONAI version 1.5.0, and CUDA version 12.4. The dataloader used six worker threads. For all VRAM and speed computations, we report the average performance from three runs.

## Results

This section presents results from training an SSL model and identifying TSS-level biases in Phikon-v2 and Inception-V4. We also present the computational performance for both LP, UMAPs, and sequential checkpointing.

### TSS classification on TCGA-LUSC

#### Linear probing TSS detection accuracy in Inception-V4 vs. pre-trained Phikon-v2.

The accuracy results from training a linear probe are in Table 2. We see a difference between Phikon-v2 and Inception-V4: Inception-V4’s LP accuracy is roughly half that of Phikon-v2. Inception-V4 is only 16% better than random guessing. The CI intervals are very narrow, suggesting the linear probe results are reproducible across different folds and that the results are based on features seen in many slides, not just a few outliers. Cohen’s κ values also support this interpretation. For Phikon-v2, κ reaches 0.51 when using all samples, representing moderate agreement beyond chance. By contrast, κ for Inception-V4 remains close to 0.15 across all settings, indicating that its small gains over chance reflect weak or inconsistent TSS separability. Together, these metrics suggest that Phikon-v2 embeddings retain coherent institution-specific structure that can be exploited even by a simple linear classifier, whereas Inception-V4 appears less sensitive to such batch effects.

**Table 2:** Linear probe accuracy scores with different numbers of samples from the dataset.

Model	Number of samples (%)	Accuracy	95% CI	κ
Phikon-v2	100	61.1	61.0–61.2	0.51
Phikon-v2	25	53.4	53.0–53.8	0.42
Phikon-v2	5	42.9	42.6–43.1	0.29
Inception-V4	100	35.6	35.5–35.7	0.19
Inception-V4	25	31.0	30.8–31.2	0.14
Inception-V4	5	29.3	29.1–29.5	0.12

[27] also used LP with Phikon-v2 on TCGA-LUSC, but with different TSSs and color normalizations on the tiles. Their accuracy ranged between 90% for non-normalized tiles to 70% for normalized tiles, showing that while color normalization has an impact, it does not prevent models from learning TSS artifacts. Our results align with their work, although our accuracy scores are lower. This could stem from differences in pre-processing, model training or data selection.

To identify the impact on the number of samples/tiles for LP, we ran the same tests, randomly selecting 25% and 5% from the training and validation data 100 times, while keeping tumor stage across the top five institutions balanced. For each test, we trained a new linear probe while keeping the test set fixed. To compensate for the lack of data, we also increased the number of epochs to 60. The results in Table 2 show that accuracy scores increase with the number of samples, but that higher higher-than-random accuracy scores are still present with only 5% of the data.

#### UMAP plots from Phikon-v2 and Inception-V4 for TSS clustering.

**Fig 6:**
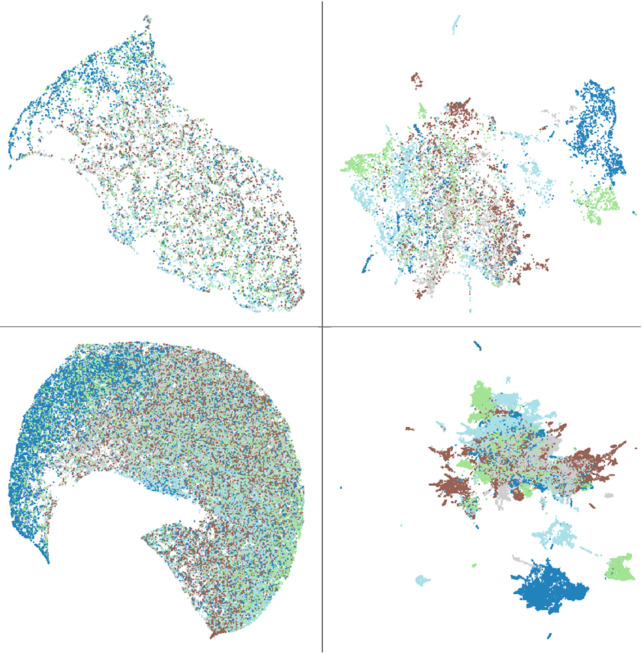
UMAP embeddings from Inception-V4 (left) and Phikon-v2 (right). The top row uses 10 000 randomly sampled tiles from the top five TSSs. The bottom row uses 70 000 points. The left and the right sides use the same points for UMAP clustering. Each colored dots represent the following TSSs: • 22 • 39 • 60 • 66 and • 85.

We identify TSS bias in a UMAP by observing distinct, non-overlapping clusters when points are color-coded by TSS. Such separation implies the model groups tiles by TSS, showing its reliance on TSS artifacts. Fig 6 shows UMAP plot embeddings from Phikon-v2 and Inception-V4. Each UMAP used the same set of tiles, randomly sampled from the top five tissue-source sites in TCGA-LUSC. We used both 10 000 and 70 000 samples to detect local and global patterns. In both examples, Phikon-v2 has the least TSS overlap. This suggests that the model is aware of and uses TSS artifacts. It also aligns with high TSS LP scores. The Inception-V4 encoder from MoCo v1 shows high TSS overlap. This suggests that the model is unaware of or does not use TSS artifacts. It is difficult to compare the plots from Inception-V4 and Phikon-v2 because the number of clusters and their structures differ. A model developer would have to consider either using a different set of points or reducing the number of points to get a clearer interpretation. To get a clearer view, our web view also allows users to filter points by tissue source site independently or by subgroup. However, in this case, it does not reveal any other obvious patterns. This also aligns with the lower LP scores.

We assessed label separation in the UMAP space using silhouette scores and K-means with Adjusted Rand Index (ARI) [67]. For the embeddings in Fig 6, all values were near zero (range: –0.07 to 0.18), indicating weak global structure. Although one might expect Inception-V4 to yield near-zero scores (substantial overlap) and Phikon-v2 to be higher, the Phikon-v2 UMAPs have multiple local clusters within each TSS. Since both silhouette scores and k-means use Euclidean distances between points, having multiple, local clusters increases the mean intra-label distances, bringing the final scores closer to zero. Similar caveats are noted in single-cell analyses [68], where the authors recommended evaluating more homogeneous subsets (specifically, similar cell types) when using the silhouette scoring function. In histology tiles, however, forming homogeneous subsets is challenging because tile-level labels are typically unavailable and tiles vary widely in cellularity and artifacts (for example, pen marks, folds), which can induce local clusters. We therefore treat silhouette and ARI as screening measures and complement them with LP-accuracy scores and qualitative inspection. For accurate interpretation using these scoring metrics, users could select similar tiles from each TSS; however, the selection of tiles will also impact the interpretation.

The reason that a foundational model such as Phikon-v2 may be more aware of TSS artifacts is that it has been trained on much larger datasets: with more data, there is less probability of having multiple tiles from the same TSS per batch. If each TSS occurs alone in a batch and has a strong site-specific artifact, the model can use these artifacts (“shortcuts”) to compare/contrast tiles. The second reason may be that larger models have many more parameters, which allows them to learn more fine-grained signatures that may include TSS-level artifacts. More research is needed to fully understand how to prevent learning shortcuts and their impact on model accuracy.

### Computational performance

#### CL model training execution time.

To better understand how sequential checkpointing impacts model training, we used various batch sizes and evaluated VRAM usage and training time. We measured VRAM usage once per second while training the model in a separate thread using the “pynvml” package version 12.0.0 [69]. We conducted our experiments with batch sizes of 32, 64, 128, and 256 during training of the MoCo v1 model using a training dataset of 189,322 tiles (similar to Table 1).

Table 3 shows the memory usage with and without sequential checkpointing. The experiments were run on both the NVIDIA 3090 and the NVIDIA L40s. The memory usage was within 200 MB of the same on both GPUs. From the table, we can see that sequential checkpointing saves between 54% and 64% memory. This also allows using a batch size of 256, which otherwise would require more VRAM.

**Table 3:** VRAM usage during training of MoCo v1, with sequential checkpointing (sc) and without. For a batch size of 128 without sequential checkpointing, only the L40S had enough memory to run. For 256, both setups were out-of-memory (OOM).

Batch Size	VRAM sc	VRAM no sc.	% MB saved with sc
32	3743	8147	54.6
64	5489	15525	64.6
128	9981	29001	64.5
256	17021	OOM	

Table 4 summarizes wall-clock training time (seconds) for MoCo-V1 over 10 epochs using two hardware platforms: an RTX 3090 and an NVIDIA L40S. We compare runs with sequential checkpointing (sc) and without checkpointing (no-sc). On the RTX 3090, enabling checkpointing produced a small reduction in wall-clock time (1.5–4.6% faster for the two batch sizes where both conditions completed), whereas on the L40S, results were effectively unchanged (differences ≤1.3%), and checkpointing enabled training at a batch size (256) that otherwise caused an out-of-memory (OOM) failure. Overall, the penalty for enabling sequential checkpointing is negligible in our setup, and in several cases, checkpointing yields a small speed-up.

**Table 4:** Time usage (seconds) for 10 epochs of MoCo-V1 training. ‘sc’ = sequential checkpointing; ‘no-sc’ = standard run. Percent differences are computed (sc – no-sc)/no-sc: negative values indicate that checkpointing reduced runtime. Blanks / N/A indicate runs that were not completed under the given configuration.

Batch size	RTX 3090 (10 epochs)		L40S (10 epochs)		%Δ (3090) (sc vs no-sc)	%Δ (L40S) (sc vs no-sc)
	sc (s)	no-sc (s)	sc (s)	no-sc (s)		
32	15,425	16,163	10,220	10,236	–4.6%	0.1%
64	15,240	15,466	10,287	10,246	–1.5%	+0.4% (slower)
128	15,019	OOM	10,163	10,293	N/A	–1.3%
256	14,958	OOM	10,194	OOM	N/A	sc: ran, no-sc: OOM

These results are consistent with recent observations that sequential checkpoints can reduce memory traffic and improve end-to-end throughput using *fusion operators* (see [70]), but they contrast with the textbook expectation of a runtime penalty for recomputation (see for example the original paper [61] or [71]). However, the near-constant wall-clock times across batch sizes may indicate that other dominant factors determine the runtime. We tried different numbers of threads for loading data (“num_workers”), but each experiment had similar run times. Our findings nevertheless indicate that sequential checkpointing is not necessarily a significant penalty to the time used for model training.

Finally, using a batch size of 256 had negligible effects on downstream tasks compared to 128, including linear probes and UMAP plots. The UMAP visualizations for different configurations were qualitatively similar, and the linear probe accuracy for TSS remained within 5% of baseline performance.

#### UMAP plot GPU acceleration.

**Fig 7:**
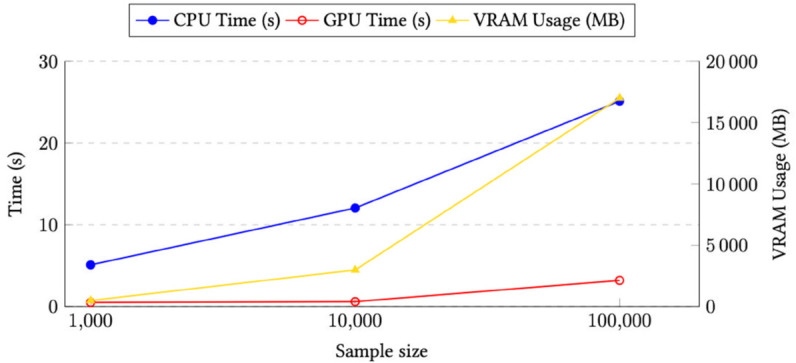
Speed & VRAM comparison UMAP on CPU vs GPU and VRAM usage for 1000, 10 000 and 100 000 points. While the speedup for 100 000 points is better on the GPU, it also requires more VRAM.

Our framework is intended for use with consumer-grade hardware. We compare existing UMAP algorithm implementations on both CPU and GPU. We evaluate both execution time and VRAM usage. Fig 7 has a comparison of CPU and GPU execution times, together with VRAM usage. We observe similar performance gains to those reported by the authors of the GPU implementation in [49]: a speedup of up to 5x with 1000 samples and 8x with 10 000 samples.

The VRAM usage for processing 100 000 embeddings extracted from tiles by Inception-V4 and Phikon-v2 is approximately 17 GB. Such a requirement may pose challenges when analyzing large datasets, such as the 460 million tiles used to train Phikon-v2. While 100 000 samples may suffice for certain analyses, optimal usage would benefit from generating multiple plots under varying sampling conditions and classes. For significantly larger datasets, users would need access to a more powerful GPU setup or rely on CPU-based implementations, which could result in rendering times up to 100 times slower.

#### Computational resources for linear probing.

Linear probing is computationally inexpensive because it requires training only a single linear layer. We trained our models for 20 epochs. TSS prediction accuracy generally did not improve beyond 20 epochs, though this may depend on the learning rate, optimizer, and other hyperparameters. [24] used five epochs and a much larger dataset sampled from 8579 slides. We have not found any other literature that discusses how many epochs are needed to achieve a good level of accuracy. Nevertheless, in our experiment, each additional epoch of linear probing added little overhead - for 67655 tiles converted to 1536-element embeddings in batches of 256 elements, each epoch added 0.34 seconds. The total time used, including validation, logging, and saving intermediate states, was approximately 30 seconds. This duration is brief enough to allow users to explore multiple options and can be incorporated into existing model training routines without adding significant delay.

## Discussion

In this work, we developed an open-source, model-agnostic framework for detecting bias and overfitting in deep learning models for whole-slide image (WSI) analysis. Our framework provides a user-friendly approach that can be used on its own or with MONAI. We also leverage cuCIM for GPU-accelerated UMAP computations. Importantly, the framework was designed to be modular and reproducible: users can swap datasets, alter sampling strategies, and add covariates without changing the core codebase. We also demonstrate how to reduce the memory footprint of larger deep learning models.

We present a methodological demonstration using a single dataset to detect tissue-submitting-site (TSS) shortcuts, rather than a comprehensive survey across datasets with multiple variables. We only use a single dataset for clarity and reproducibility: this controlled setup reduces confounding sources, keeps the presentation compact, and lets us focus on implementation details (VRAM, wall-clock time, and API ergonomics) that are central to adoption. We acknowledge that validating the framework’s generality requires running the same analyses across different types of data and other potentially confounding variables (such as age, sex, scanner type, stain protocol) and that some confounders - particularly slide- or patient-level effects- may be hard to inspect without large per-class sample sizes, although our preliminary findings such that roughly 200 samples per TSS for five TSSs may yield sufficient results - at least for LP. The framework is intentionally modular and lightweight, so that such extensions are straightforward; we therefore present this work as a usability-first tool and invite follow-up studies that apply it in broader settings with additional datasets.

It may be that UMAP plots show high clustering and that linear probes achieve high accuracy even though the underlying model is not biased or overfitted. This paper carefully selects only the top five TSSs and creates a balanced dataset with respect to tumor staging. Without doing this, one of the TSSs may have had a higher percentage of patients with advanced cancer, and another may have had a majority of healthy patients, giving an illusion of bias even though the model is accurately separating tumor staging. Without quality control, there may also be obvious artifacts in a TSS, such as blurring from an improperly configured scanner. The model may learn to recognize these in an individual TSS, without necessarily being biased or overfitted to normal data. Therefore, our tools serve mostly as an inspection or debugging tool, but it is necessary to back up those claims with the data. [23], for example, analyzed the color profiles of different TSSs in TCGA to confirm the differences in slides from different TSSs.

Interpreting UMAP plots poses challenges due to information loss during dimensionality reduction, non-uniform feature representations, and parameter sensitivity. Although UMAP interpretation depends on the viewer, the method is scalable to large datasets and provides an intuitive interface for exploring data anomalies and clustering patterns. The benefit of UMAP analysis is the ability to analyze large clusters of data. Other methods, such as saliency maps, allow users to inspect what users see in individual images: we have included samples in S1 Appendix.

The TSS-artifacts used by foundational models underscore a critical challenge in model evaluation: balancing the trade-off between high accuracy and potential bias. Similar dilemmas are observed in other areas of deep learning, such as adversarial robustness, where models are trained on noisy images to enhance generalizability [72], often at the cost of prediction accuracy. To consider the balance between bias and clinical accuracy, we would also have to consider the many clinical tasks available (such as tumor stage prediction, survival analysis or tissue classification), as well as generating tile-level labels or aggregating tile-features for slide-labels (for example gaussian mixture models [73] as in [21] or gated MIL attention models [74] used in [75]). We emphasize, however, that clinical validation should always consider a balance between accuracy and robustness to distributional shifts, as well as potential misuse of artifacts.

When bias or overfitting is detected, several mitigation strategies are available. If retraining is feasible, TSS-aware data partitioning that enforces TSS separation across training and evaluation folds has been shown to reduce TSS-level biases in downstream models [23]. For contrastive learning approaches (for example MoCo v1), conditional sampling can be used to ensure that each mini-batch contains samples from multiple TSSs, thereby discouraging the learning of site-specific shortcuts [21]. When retraining is not possible or desirable, post-hoc or preprocessing techniques may be used; for example, color normalization has been proposed to attenuate TSS-related artifacts [76], and feature-space pruning can suppress sensitive attributes in learned representations [77]. The choice among these methods depends on model class, available computational resources, and the specific bias to be addressed. Our framework supports quick and systematic exploration of such factors (for example TSS, staining, scanner) and enables sensitivity analyses to guide the selection of appropriate mitigation strategies.

We demonstrate that sequential checkpointing not only enables the use of larger models but can also support increased batch sizes. In our setup, the time cost of adding sequential checkpoints was negligible. This improvement makes it practical to use SSL models for training and fine-tuning on consumer-grade hardware, broadening their accessibility. Our improvements may be specific to MoCo v1 and our hardware. However, our results encourage more exploration for other developers to find optimal settings to run models on memory-constrained computers.

Evaluating UMAP plots and LP requires converting image data into embeddings via model inference, which can be time-intensive with large sample sizes and may take several minutes. To address this problem, we optimize runtime by integrating LP and UMAP evaluations into the training process, allowing us to cache/reuse embeddings. Inference speed can be improved by using compressed models or reducing the embedding dimensionality, though further research is needed to understand how this affects UMAP and LP results.

## Conclusion

We introduce an open-source framework that combines UMAP embeddings, cluster scoring algorithms, and linear probing to systematically detect bias and overfitting in WSI models. The framework has been tuned to run on consumer-grade GPUs by leveraging sequential checkpointing, lowering the barrier to large-scale model debugging. Applying these tools, we find that foundation models such as Phikon-v2 often encode tissue-submitting-site (TSS) signatures that can reduce cross-site generalizability. Contrastive models trained on a single, well-curated dataset showed less TSS signal in our experiments, though foundation models frequently retain advantages for other downstream tasks. Crucially, UMAP-based inspection is exploratory and can be confounded by heterogeneous tile characteristics (such as cellularity and tissue type), staining artifacts, patient covariates (such as age, sex, stage, and scanner), and parameter selection. Future work should (1) improve objective UMAP scoring (to find relevant scoring functions relative to the data distribution), (2) formalize covariate-aware selection of data/tiles, and (3) evaluate downstream impacts on segmentation and clinical tasks across external cohorts. These steps will help balance model robustness and task performance in computational pathology.

## Supporting information

S1 Appendix Saliency maps from Inception-V4 and Phikon-V2. (PDF)
