# Supplementary material for: Open-source framework for detecting bias and overfitting for large pathology images
Source: PLoS One. 2026 Feb 19;21(2):e0341715. doi: 10.1371/journal.pone.0341715 (PMC12919778; doi:10.1371/journal.pone.0341715)
Supplement: S1 Appendix — (PDF) [file pone.0341715.s001.pdf]

S1 Appendix

Saliency maps from Inception-V4 and Phikon-V2

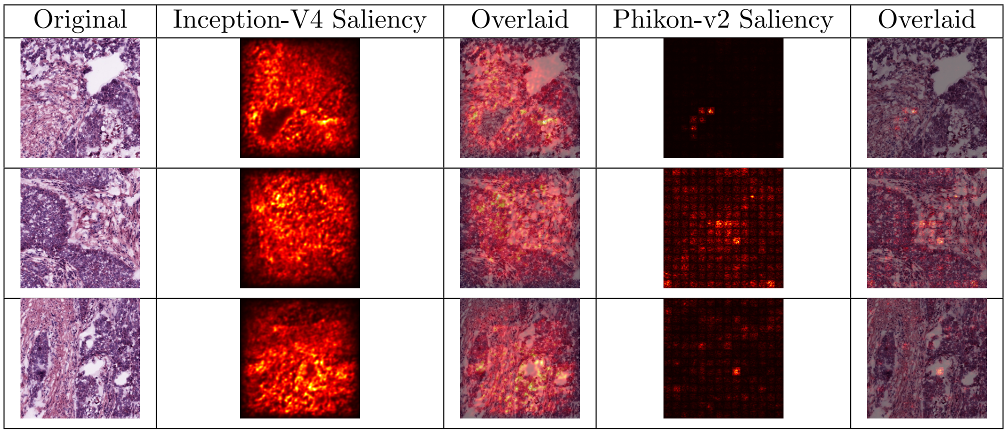

Fig S1. Saliency maps from Inception-V4 and Phikon-v2: A comparison of saliency maps using three randomly sampled tiles from TCGA-LUSC.

Figure S1 shows saliency maps computed using MONAI's "GuidedBackpropGrad". Phikon-v2 uses multiple patches per image, which explains the saliency maps' tessellated appearance. In the top image, the models seem to have an inverse attention span: the region most relevant to Phikon-v2 is not relevant to Inception-V4. For both the top and bottom images, the regions considered important for Phikon-v2 are smaller than Inception-V4, which makes it easier to interpret what features are important, suggesting the model is able to find specific markers, relevant or not, for downstream classification. Whether these differences stem from the training data or the model architecture itself is unclear without further investigation.
